# A comparative analysis of acellular versus red cell-based subnormothermic machine perfusion in human kidney transplantation

**DOI:** 10.1016/j.ajt.2026.04.014

**Published:** 2026-11

**Authors:** Harry V.M. Spiers, Michael L. Nicholson, Sara Deffrennes, Serena MacMillan, Anna L. Paterson, Miguel Larraz, Irina Mohorianu, Vasilis Kosmoliaptsis, Sarah A. Hosgood

**Affiliations:** 1 Department of Transplantation, Addenbrooke’s Hospital, Cambridge, UK; 2 Department of Surgery, University of Cambridge, Cambridge, UK; 3 NIHR Blood and Transplant Research Unit in Organ Donation and Transplantation at the University of Cambridge, Cambridge, UK; 4 Department of Development and Regeneration, Katholieke Universiteit Leuven, Leuven, Belgium; 5 Department of Nephrology, Dialysis and Renal Transplantation, University Hospitals Leuven, Leuven, Belgium; 6 Department of Histopathology, Addenbrooke’s Hospital, Cambridge, UK; 7 Cambridge Stem Cell Institute, University of Cambridge, UK; 8 Institute of Metabolic Sciences, University of Cambridge, UK

**Keywords:** normothermic machine perfusion, subnormothermic machine perfusion, acellular perfusate, RNAseq

## Abstract

Normothermic machine perfusion (NMP) typically uses red blood cell (RBC)-based perfusates, which are associated with logistical challenges. Subnormothermic machine perfusion (SMP, 32 °C) may reduce metabolic demands while preserving organ viability, but the effect of perfusate type under these conditions remains unclear. In a paired experimental design, kidneys from 8 deceased donors were randomized 1:1 to 6 hours of SMP with RBC-based (RBC-SMP) or acellular perfusate (SNAP), followed by 4 hours of RBC-based NMP (37 °C) to simulate reperfusion. Upregulation of transcriptomic ischemia-reperfusion injury-related pathways and downregulation of oxidative phosphorylation at 6 hours of SMP and 4 hours of reperfusion were similar between RBC-SMP and SNAP kidneys. No differences were observed between groups in transcriptional profiles, metabolic pathway scores, urinary tubular injury biomarker concentrations, or histology. We integrated publicly available NMP transcriptomic data for comparison, demonstrating that compared with 6 hours of RBC-NMP at 37 °C, both SMP groups showed equivalent proinflammatory pathway activation and oxidative phosphorylation depletion. Thus, we provided evidence that SNAP maintains kidneys with equivalent transcriptional and urinary tubular injury biomarker profiles to RBC-SMP. These findings support the feasibility of SNAP for clinical translation in future studies.

## 1 Introduction

Machine perfusion (MP) of deceased-donor kidneys provides advantages compared with static cold storage (SCS) as a method of organ preservation, offering potential for improved clinical outcomes and increased utilization of organs that may have been previously discarded.^1^^,^^2^ Normothermic MP (NMP) allows maintenance of organs in a near physiological state and can facilitate organ assessment, repair, and regeneration.^3^, ^4^, ^5^, ^6^ The feasibility of NMP in clinical kidney transplantation has been confirmed at the randomized controlled trial level, albeit without demonstrating superiority in clinical outcomes compared with traditional SCS.^7^ Accordingly, unlike in liver transplantation, kidney NMP has not seen widespread clinical implementation, despite renal transplantation representing the most performed solid organ transplant procedure.

NMP typically employs a red blood cell (RBC)-based perfusate to deliver oxygen and nutrients during perfusion.^3^^,^^8^ However, RBC-based perfusate carries logistical challenges, including the requirement for blood type compatibility, infection risk, immunogenicity, and hemolysis during perfusion.^9^, ^10^, ^11^, ^12^ Cell-free, or acellular, perfusates could overcome these challenges, enhance safety, and improve regulatory compliance for widespread implementation of NMP in the clinical setting. As such, there has been an increasing number of studies exploring the use of acellular perfusate for kidney perfusion across a range of temperatures in recent years, including up to 24 hours.^13^, ^14^, ^15^, ^16^, ^17^ Two recent studies have successfully transplanted human kidneys following short periods (2 hours) of acellular perfusion.^18^^,^^19^

Studies investigating NMP (at 37 °C) without the addition of an oxygen carrier show conflicting results,^16^^,^^20^^,^^21^ prompting us to perfuse kidneys with a small reduction in perfusion temperature to 32 °C, reducing the oxygen requirements of the kidney but still maintaining a high level of metabolism.^13^ Currently, there are no head-to-head comparisons between acellular vs RBC-based kidney MPs at subnormothermic (32 °C) temperatures. The aim of this study was to investigate the hypothesis that subnormothermic perfusion with an acellular perfusate is noninferior to perfusion with RBC-based perfusate. If proven, this would represent an important milestone in advancing the field. To address this, we used a same-human-donor, paired kidneys (n = 8) experimental design to perfuse kidneys with either RBC or acellular perfusion solution during subnormothermic MP (SMP), followed by a normothermic reperfusion phase using RBC-based perfusion to mimic revascularization in the recipient. We used untargeted bulk RNA sequencing transcriptomics to examine gene expression changes between perfusate groups, additionally exploring urinary markers of tubuloepithelial cell injury. We then functionally integrated publicly available bulk RNA sequencing data sets to explore transcriptomic differences between kidneys undergoing NMP and SMP with RBC and acellular perfusates to provide insights into proinflammatory pathway induction in different MP contexts.

## 2 Materials and methods

### 2.1 Study population

Sixteen human donor kidneys from 8 deceased donors were recruited into this study after being declined for transplantation and consented for research. Written consent for the use of kidneys for research was given by the donor families and was obtained by specialist nurses in organ donation. Ethical approval was obtained from the National Research Ethics Service: 22/WA/0167.

### 2.2 Study design

Paired human kidneys declined for transplantation (both kidneys from the same donor, n = 8 pairs) were randomly assigned to undergo acellular or RBC-based MP at 32 °C for 6 hours. Following 6 hours of SMP, both kidneys were “reperfused” using an RBC-based perfusate at normothermia (37 °C) to mimic implantation and reperfusion in a transplant setting (Supplementary Fig. S1).

### 2.3 Machine perfusion

All kidneys were received on ice at 4 °C after a period of SCS, prepared for MP, and weighed. Kidneys were flushed with 500 mL of cold Ringer’s solution via the renal artery. The ureter was cannulated prior to perfusion. All kidneys were perfused for 6 hours at 32 °C using an adapted pediatric cardiac bypass system (Medtronic, Biconsole 560), then were removed from the circuit and flushed with 1 L cold Ringer’s solution via the renal artery before perfusion at 37 °C for a further 4 hours using a fresh circuit. For all perfusions, the perfusion solution was oxygenated (95% O_2_/5% CO_2_) at a flow rate of 0.1 L/min, and perfusion was undertaken continuously through the renal artery.

Perfusate composition is outlined below in the Table.

**Table:** Composition of RBC-based and acellular perfusates.

Composition	RBC-based perfusate	Acellular perfusate
RBC	200 mL	-
HAS 5%	250 mL	250 mL
Ringer’s solution	100 mL	300 mL
Dexamethasone	6.6 mg	6.6 mg
Calcium gluconate 10%	5 mL	5 mL
Sodium bicarbonate 8.4%	20 mL to adjust pH	12 mL to adjust pH
Creatinine	0.055 g	0.055 g
Meropenem	250 mg	250 mg
Verapamil	1 mg	1 mg
Supplements		
Ringer’s solution	To replace urine output in mL/mL	To replace urine output in mL/mL
Synthamin 17 10% with 15 mL sodium bicarbonate 8.4% 100 IU insulin and 5 mL Cerenvit	10 mL/h	10 mL/h
Glucose 5%	3 mL/h	3 mL/h
GTN 10 mg in 100 mL	10 mL/h for 3 h then 5 mL/h	10 mLl/h for 3 h then 5 mL/h

GTN, glyceryl trinitrate; RBC, red blood cell; HAS, human albumin solution.

### 2.4 Arterial and venous oxygen content, oxygen consumption, and extraction ratio calculations

The oxygen solubility coefficient is corrected for temperature, with 0.023 used at 32 °C and 0.021 at 37 °C.

#### 2.4.1 Red cells at 32 °C

Arterial oxygen content CaO_2_ (mL/dL) = (1.34 [mL O_2_/g Hb] × Hb [g/dL] × SaO_2_ × 0.01) + (0.023 [mLO_2_/kPa/dL] × PaO_2_ [kPa])

Venous oxygen content CvO_2_ (mL/dL) = (1.34 [mL O_2_/g Hb] × Hb [g/dL] × SvO_2_ × 0.01) + (0.023 × PvO_2_ [kPa])

#### 2.4.2 Acellular at 32 °C

Arterial oxygen content CaO_2_ (mL/dL) = 0.023 (mL O_2_/kPa/dL) × PaO_2_ (kPa)

Venous oxygen content CvO_2_ (mL/dL) = 0.023 (mL O_2_/kPa/dL) × PvO_2_ (kPa)

#### 2.4.3 Red cells at 37 °C

Arterial oxygen content CaO_2_ (mL/dL) = (1.34 × Hb × SaO_2_ × 0.01) + (0.021 × PaO_2_)

Venous oxygen content CvO_2_ (mL/dL) = (1.34 × Hb × SvO_2_ × 0.01) + (0.021 × PvO_2_)

#### 2.4.4 Oxygen consumption and extraction ratios for all

Oxygen delivery DO_2_ (mL/min) = (CaO_2_ × 0.01) × flow rate

Oxygen consumption VO_2_ (mL O_2_/min/100 g) = (CaO_2_ – CvO_2_) × 0.01 × flow rate

Oxygen extraction ratio (OER) = VO_2_/DO_2=_

### 2.5 Supplementary methods

For all remaining methodology, please see Supplementary Methods.

## 3 Results

### 3.1 Hemodynamic, oxygenation, and urinary parameters during SMP

We perfused 8 pairs of human kidneys procured for transplantation but subsequently declined and offered for research. Donors had a median age of 65 (range, 45-75) years, a median body mass index of 24.77 (range, 22.76-32.87) kg/m^2^, were predominantly male (57%), and were mostly from brain stem death donation donors (71%). The median cold ischemia time was 28.2 (range, 13.4-29.6) hours. Full demographic characteristic data are presented in the Supplementary Table.

Kidneys were randomized 1:1 to undergo 6 hours of either SMP with RBC-based perfusate (RBC-SMP) or SMP with acellular perfusate (SNAP). Following SMP, all kidneys underwent “reperfusion” using RBC-based NMP at 37 °C for 4 hours (Supplementary Fig. S1). Renal blood flow (RBF) in SNAP kidneys increased significantly during perfusion (Fig. 1A), rising from a mean of 120.2 mL/min/100 g at the start of perfusion to a mean of 194.9 mL/min/100 g by 6 hours (*P* = .0037). In kidneys perfused with RBC-SMP, there was no significant change in RBF across the duration of perfusion (91.11-131.8 mL/min/100 g, *P* = .6566). There were no significant differences in RBF between RBC-SMP and SNAP cohorts at any time point (Fig. 1A). Oxygen consumption was similar in both cohorts throughout perfusion (Fig. 1B); however, the oxygen extraction ratio was significantly higher in SNAP kidneys after 1 hour of perfusion (Fig. 1C). Taken together, these data suggest SNAP provides adequate tissue oxygen delivery through increased extraction of oxygen compared with RBC-SMP. Urine production during SMP was not significantly different between groups at any time point (Fig. 1D), nor was the osmolarity of the urine produced (Fig. 1E). During the reperfusion phase, both kidney groups underwent RBC-based NMP at 37 °C. RBF, oxygen consumption and extraction, urine output, and osmolality were not different between SNAP and RBC-SMP kidneys during the reperfusion phase (Fig. 1F-J).

**Figure 1:**
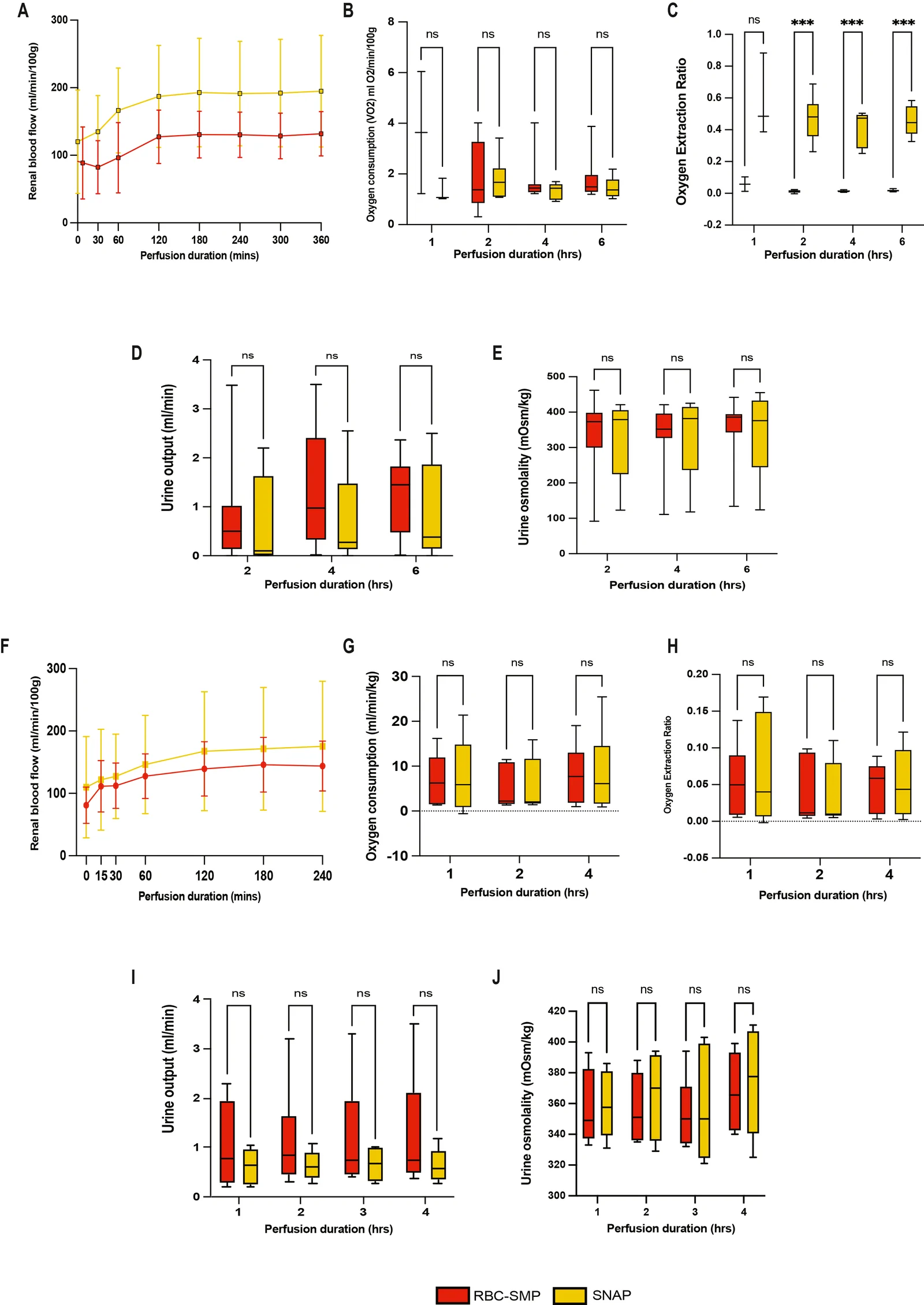
Renal blood flow (A), oxygen consumption (B) and extraction ratio (C), urine output (D), and osmolality (E) during subnormothermic machine perfusion. Renal blood flow (F), oxygen consumption (G) and extraction ratio (H), urine output (I) and osmolality (J) during reperfusion with red blood cell-based perfusion at 37 ^°^C. RBC-SMP, RBC-based subnormothermic machine perfusion; SNAP, subnormothermic acellular perfusion.

### 3.2 Transcriptional pathways during SMP are similar between RBC-SMP and SNAP kidneys

Kidneys within each donor pair demonstrated transcriptional concordance at the pre-NMP timepoint with no significantly differentially expressed genes, indicating low intra-donor biological variability and supporting the paired experimental design (Supplementary Fig. S2A). We then investigated tissue transcriptional changes between the 2 perfusate groups at the end of 6 hours of SMP, and at 4 hours of simulated reperfusion, comparing these to gene expression at the end of SCS (pre-SMP) in our paired kidney experimental design. Principal component analysis (PCA) revealed that the largest source of transcriptional variation was between biopsies taken in the cold (pre-SMP) vs in the warm (SMP and reperfusion timepoints; PC1: 39.76%), highlighting the significant, but anticipated, transcriptional changes upon reperfusion with a warm oxygenated solution following a period of cold preservation (Fig. 2A). In the top 50 genes accounting for this variation, on PCA loadings, we found transcripts for proinflammatory cytokines (*IL1B, IL6,* and *CXCL8*); coagulation pathway modulators (*SERPINE1, PLAU,* and *TFPI2*); activated endothelial cells (*SELE* and *HBEGF*); oxidative stress and apoptosis regulators (*MT1A* and SLC7A11); and pro- (*MYC* and *FOSL1*) and anti-inflammatory (*ATF3*) transcription factors (Fig. 2B). Overrepresentation analysis of these genes highlighted pathways related to immune activation and inflammatory responses, including cytokine signaling, apoptosis, and coagulation (Fig. 2C), all known to be upregulated in ischemia-reperfusion injury (IRI).^22^, ^23^, ^24^ PCA also highlighted variation in gene expression between sex groups (PC2, 20.04%) at all time points (Fig. 2D), prompting us to account for this as a covariate in our differential expression analysis. Differential gene expression at 6 hours of SMP showed that only 1 gene was significantly different between SNAP and RBC-SMP groups, namely, the β hemoglobin gene *HBB*, which was downregulated in SNAP kidneys (Fig. 2E). These results highlight transcriptional similarity at the tissue level between the 2 groups, regardless of the perfusate method used during MP.

**Figure 2:**
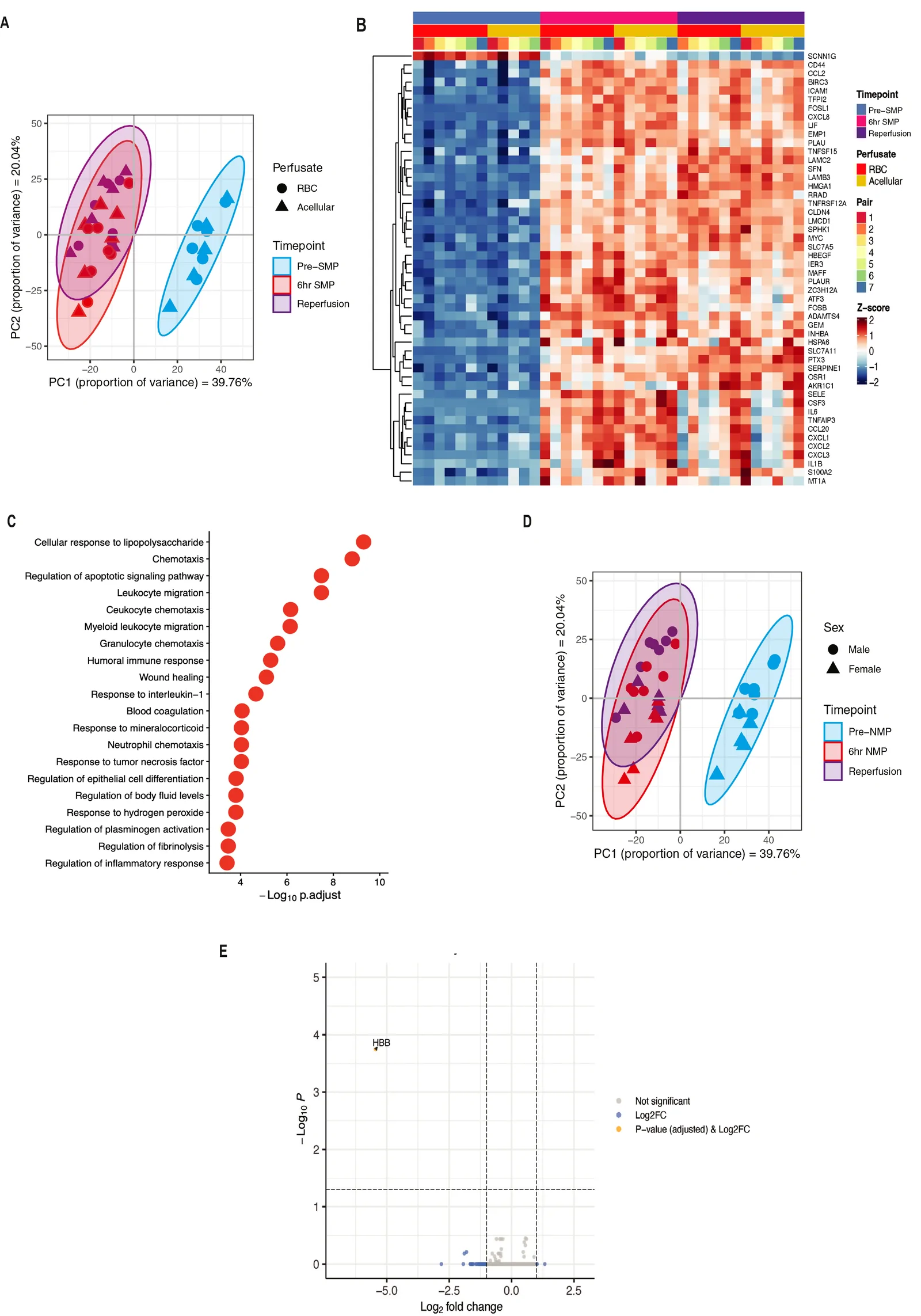
(A) Assessment of gene expression by principal component analysis (PCA) using the top 500 most variable genes. (B) Heatmap depiction of the top 50 most variable genes from the PC1 loading. (C) Reactome pathways annotated from genes in B. (D) PCA from A annotated for donor sex. (E) Volcano plot demonstrating only one significantly differentially expressed gene between paired RBC-SMP and SNAP kidneys after 6 hours of SMP. RBC-SMP, RBC-based subnormothermic machine perfusion; SNAP, subnormothermic acellular perfusion.

### 3.3 Changes in proinflammatory pathway activation in the kidneys during SMP are independent of the perfusate used

We next sought to explore the potential impact of the different perfusion methods on global transcriptomic pathway changes during SMP and at 4 hours of simulated reperfusion. Gene set enrichment analysis (GSEA) revealed enrichment of proinflammatory/immune activation and repair/proliferation pathways after 6 hours of SMP (Fig. 3A). Enriched pathways included *TNFα* signaling via *NFκB*, allograft rejection, inflammatory response*,* and complement, all of which are known to be central in kidney IRI during MP at normothermia.^22^^,^^23^ Gene sets relating to cytokine/chemokine signaling were also enriched, including *IL6/JAK/STAT3, IL2/STAT5* signaling, and interferon gamma response*.* Additionally, there was upregulation of genes annotated to pathways indicative of cell proliferation (*MYC targets)* and epithelial-mesenchymal transition. Enrichment of these pathways was observed to be of similar magnitude between RBC-SMP and SNAP cohorts, both at 6 hours of SMP and at 4 hours of reperfusion. Furthermore, in both perfusate groups, there was downregulation of the oxidative phosphorylation gene set at the end of SMP and at reperfusion, compared with the end of SCS.

**Figure 3:**
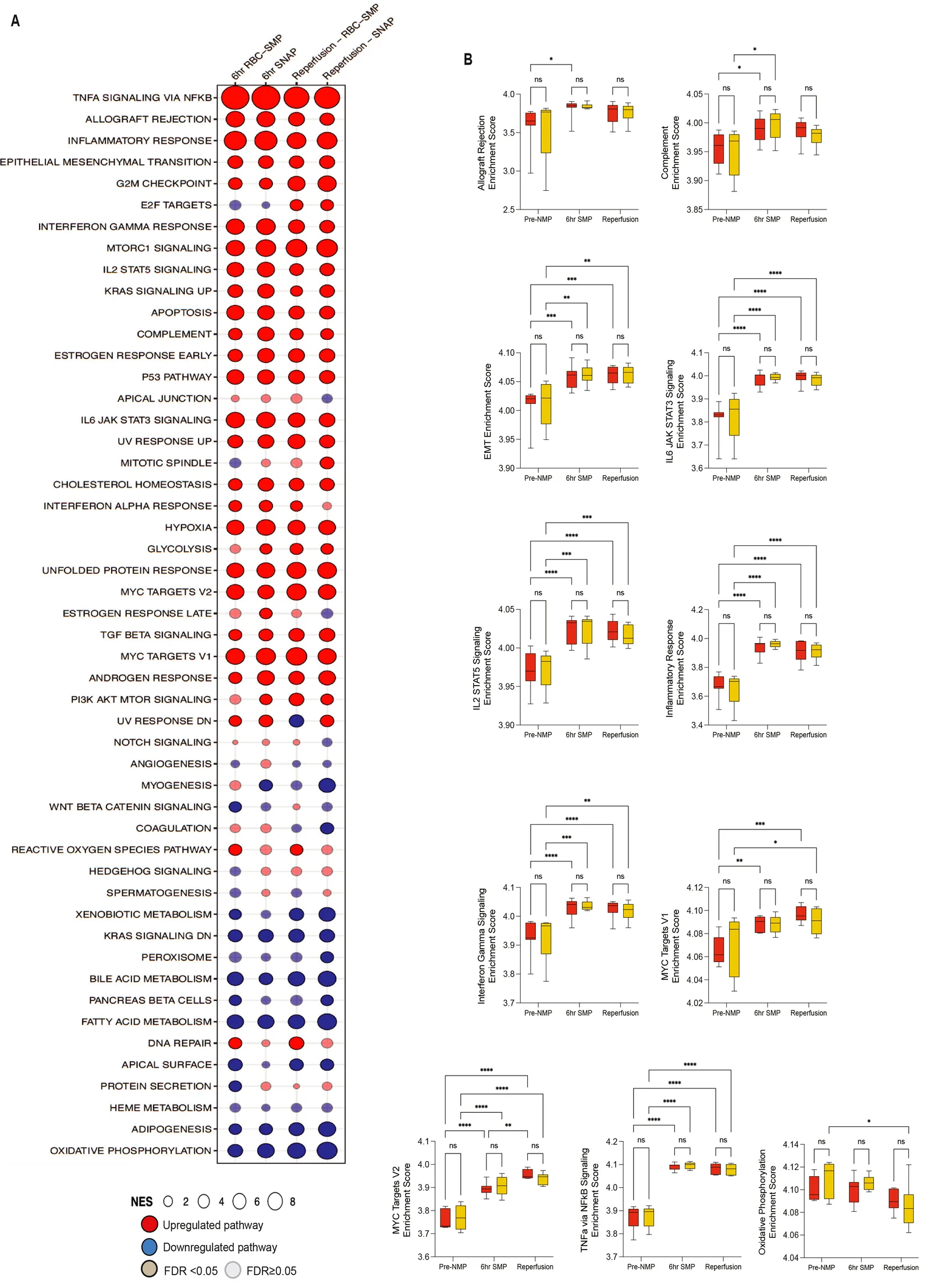
(A) GSEA against the hallmarks pathways using differentially expressed genes between the pre-SMP timepoint biopsy and the annotated timepoint. (B) Log2 transformed ssGSEA scores and comparisons for select pathways; red represents RBC-SMP and yellow SNAP. FDR, false discovery rate; GSEA, gene set enrichment analysis; RBC-SMP, RBC-based subnormothermic machine perfusion; SNAP, subnormothermic acellular perfusion; ss GSEA, single-sample gene set enrichment analysis.

Having demonstrated similar patterns of global gene enrichment between SNAP and RBC-SMP cohorts, we used single-sample GSEA to quantify differences between perfusion groups, both at the end of SMP and at 4 hours of simulated reperfusion. We found an increase in the enrichment scores for the aforementioned proinflammatory/immune activation pathways between SCS and the 6-hour SMP timepoint; however, there were no statistically significant differences in the magnitude of that enrichment between RBC-SMP and SNAP kidneys (Fig. 3B). The same was true for group comparisons at the reperfusion phase, with no significant differences between perfusion cohorts. This suggests the perfusate method had no differential effect on the magnitude of the primary IRI affecting all kidneys during SMP, or on the magnitude of the secondary IRI during the simulated reperfusion phase. Interestingly, although single-sample GSEA confirmed no significant differences, at any time point, between RBC-SMP and SNAP kidneys, within-group comparisons for the oxidative phosphorylation pathway demonstrated greater pathway downregulation within SNAP kidneys between SCS and reperfusion time points (Fig. 3B).

We next sought to explore how SMP compares to NMP at the global transcriptional level. We capitalized on publicly available bulk transcriptomic data from the study by de Haan et al^11^ to explore transcriptional signatures induced after RBC-NMP at 37 °C. GSEA of differentially expressed genes at 6 hours of RBC-NMP, RBC-SMP, and SNAP (compared with biopsies at SCS) revealed induction of similar proinflammatory and immune activation pathways associated with kidney IRI,^22^^,^^23^ including *TNFα* via *NFκB* signaling, inflammatory response, allograft rejection and *IL6 JAK STAT3* signaling (Supplementary Fig. S3). This enrichment was of similar magnitude in all groups, suggesting that lowering the temperature to 32 °C does not mitigate IRI, regardless of RBC-based or acellular perfusate. Notably, depletion of the oxidative phosphorylation pathway was seen at the 6-hour time point in all MP groups. Taken together, these data suggest that kidneys experience a similar magnitude of IRI at 6-hour NMP and SMP.

### 3.4 Metabolism-associated transcriptional pathways show no significant differences between acellular and RBC-based perfusate

A cell-free nutrient-supplemented perfusate has been demonstrated to facilitate extended ex vivo preservation of metabolically active kidneys.^17^ Transcriptomic pathway analysis has been previously used to gain metabolic insights into kidney physiology.^25^ Based on this precedent, we leveraged our transcriptomic approach to explore changes in several relevant metabolic pathways in kidneys undergoing RBC-SMP and SNAP (see Transcriptomic Pathway Activity Scoring; Materials and methods section). We found that for all pathways explored, there was no statistically significant difference between RBC-SMP and SNAP kidneys at any time point, suggesting that an acellular perfusate can support kidney metabolic functions during 6 hours of SMP (Fig. 4). RBC-SMP kidneys demonstrated an increase in glycolysis scores during SMP and at reperfusion, compared with pre-SMP, whereas SNAP kidneys showed no statistically significant difference (Fig. 4A). The tricarboxylic acid (TCA) score gradually reduced during SMP and at reperfusion for RBC-SMP kidneys but was only reduced at reperfusion (compared with the pre-SMP timepoint) in the SNAP cohort (Fig. 4B). In addition to glucose, glutamine is an important nutrient source for glycolysis and the TCA cycle. In both groups, glutamine synthesis scores significantly increased during SMP, with a trend toward further increase at reperfusion, potentially reflecting utilization and depletion of this cellular energy source in the period of ischemia inherent to organ procurement and transport on SCS, followed by repletion during SMP and during reperfusion (Fig. 4C). Fatty acid oxidation pathway scores were seen to be reduced in both groups at 4 hours of reperfusion (Fig. 4D), with additional reduction in gluconeogenesis scores (Fig. 4E); coupled with enhanced glycolysis pathway scores throughout perfusions, this may reflect metabolic reprogramming to compensate for energy shortage during acute kidney injury. Polyol metabolism converts glucose to fructose via sorbitol, typically in hyperglycemia but also under normoglycemia in ischemia, inducing osmotic and oxidative stress; it has been implicated in tubular cell injury during IRI.^26^^,^^27^ In both cohorts, polyol metabolism scores reduced during SMP with a further significant reduction at reperfusion (Fig. 4F), potentially reflecting restoration of canonical glucose metabolism during oxygenated perfusion.

**Figure 4:**
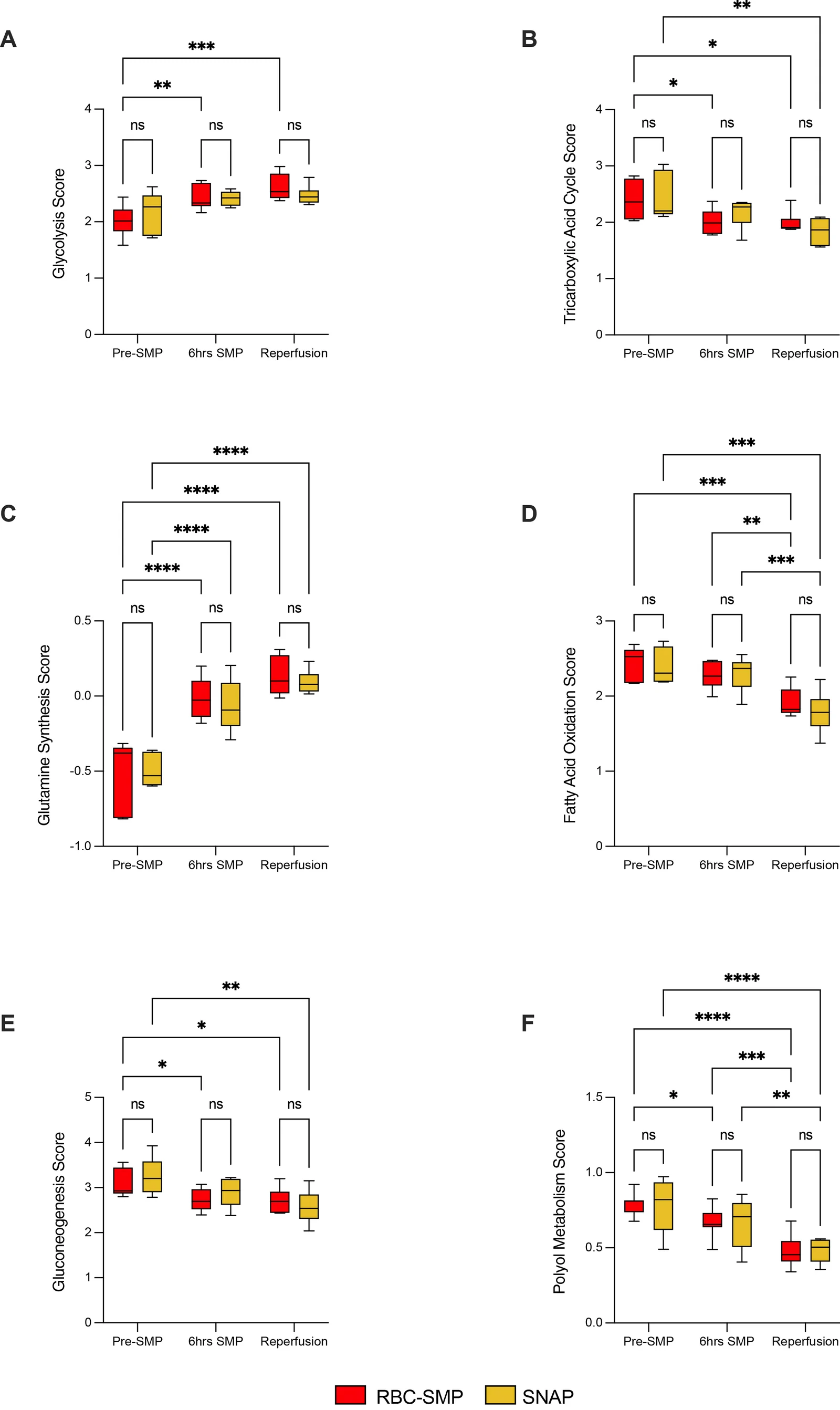
Metabolic pathway scores for glycolysis (A), tricarboxylic acid cycle (B), glutamine synthesis (C), fatty acid oxidation (D), gluconeogenesis (E), and polyol metabolism (F). FDR, false discovery rate; NES, normalized enrichment score; RBC-SMP, RBC-based subnormothermic machine perfusion; SNAP, subnormothermic acellular perfusion.

### 3.5 Urinary biomarker levels of tubuloepithelial cell injury are similar in both RBC-SMP and SNAP perfused kidneys

In homeostasis, there is a significant gradient in oxygen pressure and osmolarity over the corticopapillary axis of the kidney, leading to increased osmotic cellular stress.^28^ We hypothesized that restoration of metabolic function during SMP may restore osmotic cellular stress. Therefore, we defined an osmotic stress score by averaging expression of related genes^25^^,^^29^ and assessed changes at 6 hours of SMP and at 4 hours of simulated reperfusion (Fig. 5A). The osmotic stress score increased in both RBC-SMP and SNAP kidneys during SMP, with no further increase at reperfusion, with no significant difference between groups, suggesting an acellular perfusate does not increase osmotic stress relative to RBC-based perfusion.

**Figure 5:**
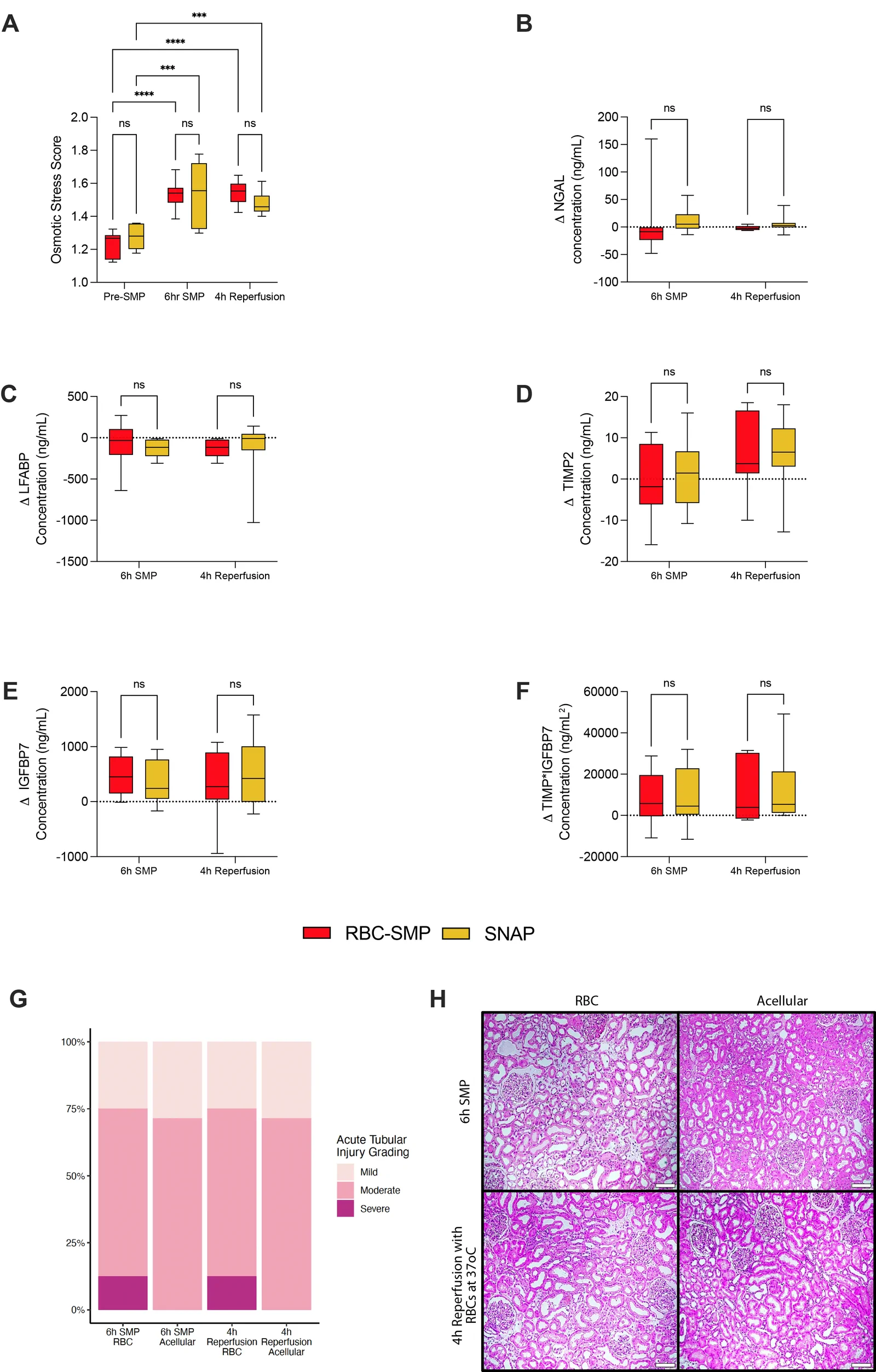
(A) Osmotic stress score across perfusion timepoints. Change in urine concentration of key urinary injury biomarkers, including NGAL (B), L-FABP (C), TIMP2 (D), IGFBP7 (E), and multiplicated TIMP2∗IGFBP7 (F). (G) Histologic assessment of acute tubular injury grading between perfusion groups. (H) Representative H&E images of kidneys at 6 hours of SMP and 4 hours of reperfusion. H&E, hematoxylin and eosin; IGFBP7, insulin-like growth factor-binding protein 7; L-FABP, liver-type fatty acid-binding protein; NGAL, neutrophil gelatinase-associated lipocalin; RBC-SMP, RBC-based subnormothermic machine perfusion; SNAP, subnormothermic acellular perfusion; TIMP2, tissue inhibitor of metalloproteinase 2.

Having identified an upregulation of osmotic stress-associated genes during SMP in both groups, we hypothesized that, in the context of proinflammatory pathway upregulation (discussed earlier), this may reflect tubuloepithelial cell injury rather than purely homeostatic osmotic stress. Therefore, we sought to explore at the protein level whether there were differences in urinary biomarker levels of tubular injury between RBC-SMP and SNAP kidneys (Fig5B-F). We selected neutrophil gelatinase-associated lipocalin,^30^, ^31^, ^32^ liver-type fatty acid-binding protein,^33^^,^^34^ tissue inhibitor of metalloproteinase 2 (TIMP2), and insulin-like growth factor-binding protein 7 (IGFBP7)^35^^,^^36^ because of their association with posttransplant outcomes or acute kidney injury during MP. Additionally, we examined concentrations of combined TIMP and IGFBP7 markers (TIMP∗IGFBP7) given their value in predicting adverse outcomes in acute kidney injury.^37^^,^^38^ We assessed the change in urinary concentration of each marker between timepoints (2 hours vs 6 hours SMP; 1 hour vs 4 hours reperfusion). There were no significant differences in the change in urinary concentration of the above urinary injury markers between RBC-SMP and SNAP kidneys during SMP or during the reperfusion phase. These findings were corroborated by histologic assessment by a blinded transplant histopathologist, which showed similar tubular injury profiles between RBC-SMP and SNAP kidneys, both during SMP and at reperfusion (Fig. 5G, H).

## 4 Discussion

Kidney MP offers advantages over SCS, but commonly used RBC-based perfusates carry logistical challenges and can drive ferroptotic tubular injury via free heme release by RBC lysis. Development of cell-free/acellular perfusates and subnormothermic perfusion (32 °C) may reduce metabolic demands and mitigate reperfusion injury. We used a paired kidney experimental design to provide the first direct comparison of RBC and acellular perfusates during SMP of human kidneys at 32 °C. We performed bulk RNA sequencing, demonstrating that the dominant transcriptomic pathway changes during SMP and at simulated reperfusion with RBCs at 37 °C were related to ischemia-reperfusion injury, including upregulation of *TNFα* via *NFκB* signaling, allograft rejection, and inflammatory response, and were not different between RBC-SMP and SNAP kidneys. Similarly, the downregulation of oxidative phosphorylation relative to SCS was comparable, with no significant modulation by perfusate type. We explored transcriptional changes in metabolic programs during SMP and at simulated reperfusion, suggesting that both RBC-SMP and SNAP provide similar support to select renal metabolic functions. We further demonstrated by histologic comparison and direct measurement of validated urinary biomarkers of acute tubular injury that SNAP did not induce more tubuloepithelial injury compared with RBC-SMP. Finally, our analysis integrating publicly available transcriptomic data suggested that RBC-based NMP induces similar transcriptomic changes compared with subnormothermic perfusion.

A key observation was the similarity in transcriptional changes associated with metabolic pathways between SNAP and RBC-SMP kidneys, with no significant differences between groups at 6 hours of SMP and at simulated reperfusion. This suggests similar patterns of changes in metabolism after cold storage when using either RBC-based or acellular perfusate. Transcription of glutamine synthesis-associated genes increased during SMP and at simulated reperfusion, suggesting replacement of this key anaplerotic substrate after ischemic depletion; prior evidence implicates glutamine in renoprotection during ischemia-reperfusion injury due to its role in intracellular redox homeostasis.^39^ Reductions in fatty acid oxidation and gluconeogenesis pathway genes at reperfusion suggested a shift toward glucose utilization as its availability was restored, while suppression of polyol metabolism implied a move away from stress- and IRI-associated glucose fluxes linked to tubular injury. Interestingly, while RBC-SMP kidneys showed a greater relative induction of glycolysis across the duration of SMP and simulated reperfusion and an earlier reduction of inferred TCA cycle activity, these kinetic differences did not alter overall transcriptional metabolic trajectories, suggesting that oxygen carrier presence is not required for the major transcriptomic features of metabolic recovery at 32 °C. The TCA cycle has been previously shown to be maintained during kidney perfusion by an acellular perfusate up to 4 days of MP^17^; the lagging changes in TCA pathway scores seen in SNAP vs RBC-SMP kidneys within our study may reflect differential metabolic maintenance of kidneys ex vivo using an acellular perfusate. Future studies combining transcriptomics with metabolomic profiling or isotope tracing are required to validate our findings at the tissue level.

We acknowledge the limitations of our study, including a modest sample size, reflecting the limited availability of paired human kidneys declined for transplantation. To mitigate this, we used a paired design, using kidneys from the same donor for both arms, which controls for interdonor variability and strengthens internal validity. However, we accept our study was not powered to assess the impact of donor heterogeneity, including donation type or cold ischemic time, on the study’s endpoints. Our transcriptomic findings would have been strengthened by validation at the protein level, and this should be included in future work. Nevertheless, we complemented the transcriptomic analysis with validated urinary biomarker levels of tubular injury, as well as histologic assessment, which provided orthogonal support for comparable IRI in RBC-SMP and SNAP kidneys. Although we have previously shown the potential for SNAP to support kidneys for up to 24 hours,^13^ the focus of this study was on end-ischemic subnormothermic perfusion after SCS for 6 hours, a clinically relevant time period during which a recipient can be brought in and prepared for transplant. Further studies are required to understand the impact of different perfusates at a more granular level and longer perfusion timepoints, including spatial localization of transcriptomic pathway activation to understand cell-specific responses. Finally, these kidneys were not transplanted, so we cannot directly assess in vivo graft function. We included a normothermic reperfusion phase to simulate implantation and did not find any differences between acellular and RBC-based perfusion methods.

Taken together, our data support that subnormothermic perfusion for 6 hours with acellular perfusate is noninferior to perfusion with RBC-based perfusate. Acellular perfusates offer logistical and potential safety advantages by avoiding the requirement for blood type matching and the risks of hemolysis and free haem toxicity. Notably, organs have successfully been transplanted following 2 hours of SNAP with controlled rewarming, achieving acceptable outcomes.^15^^,^^18^^,^^19^ Our findings further support the translation of SNAP through future studies assessing the safety and efficacy of longer periods of SNAP in the clinical setting.

## Data availability

The data sets presented in this study can be found in online GEO accession repositories: https://www.ncbi.nlm.nih.gov/geo/ (Gen number: GSE343974).

## Funding

The author(s) declare that financial support was received for the research and/or publication of this article. We acknowledge funding support from the National Institute for Health and Care Research (NIHR) Blood and Transplant Research Unit in Organ Donation and Transplantation (NIHR203332), a partnership between NHS Blood and Transplant, University of Cambridge, and Newcastle University. HS acknowledges funding from a Sir Roy & Lady Calne Royal College of Surgeons Research Fellowship (G121988) and a Wellcome Trust Clinical Research Training Fellowship (G115288). IM acknowledges funding from Wellcome Trust (203151/Z/16/Z) and UKRI Medical Research Council (MC_PC_17230). VK acknowledges funding from an NIHR Fellowship (PDF-2016-09-065) and as a Paul I. Terasaki Scholar (G106170).

## Declaration of competing interests

The authors of this manuscript have no conflicts of interest to disclose as described by *American Journal of Transplantation*.

## Appendix A Supplementary data

Multimedia component 1

Supplementary Figure 1 A: Schematic of experimental design demonstrating subnormothermic machine perfusion phase and 4 hours reperfusion with RBC-based perfusate at 37oC. Figure produced using Biorender.

Supplementary Figure 2 A: Volcano plot demonstrating no significantly differentially expressed genes between paired RBC-SMP and SNAP kidneys at the end of cold storage, prior to SMP. Dashed line represents significance level of p<0.05.

Supplementary Figure 3 A: GSEA against the Hallmarks pathways using differentially expressed genes between pre-perfusion timepoint biopsy and 6 hour timepoint biopsy for the three perfusion types. Raw GSEA output data available in Supplementary Table 2. RBC-SMP, RBC-based subnormothermic machine perfusion; SNAP, subnormothermic acellular perfusion; RBC-NMP, RBC-based normothermic machine perfusion; NES, normalized enrichment score; FDR, false discovery rate.
