# Supplementary material for: A comparative analysis of acellular versus red cell-based subnormothermic machine perfusion in human kidney transplantation
Source: Am J Transplant. 2026 Nov;26(11):2511–23. doi: 10.1016/j.ajt.2026.04.014 (PMC13768858; doi:10.1016/j.ajt.2026.04.014)
Supplement: Supplementary file 1 [file mmc2.pdf]

A

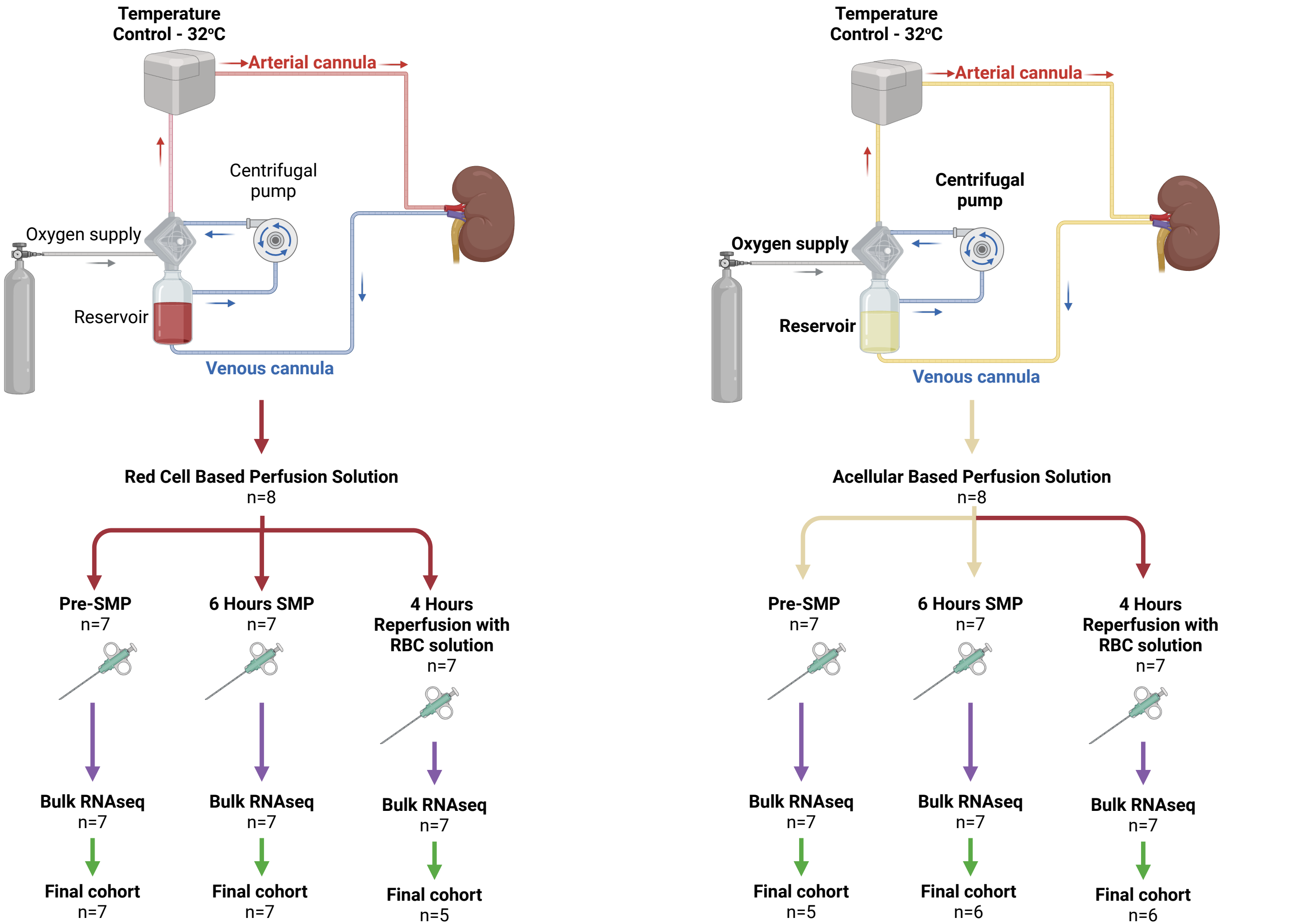

Temperature
Control - 32°C
Arterial cannula
Centrifugal
pump
Oxygen supply
Reservoir
Venous cannula
Red Cell Based Perfusion Solution
n=8
Pre-SMP
n=7
6 Hours SMP
n=7
4 Hours
Reperfusion with
RBC solution
n=7
Bulk RNAseq
n=7
Bulk RNAseq
n=7
Bulk RNAseq
n=7
Final cohort
n=7
Final cohort
n=7
Final cohort
n=5
Temperature
Control - 32°C
Arterial cannula
Centrifugal
pump
Oxygen supply
Reservoir
Venous cannula
Acellular Based Perfusion Solution
n=8
Pre-SMP
n=7
6 Hours SMP
n=7
4 Hours
Reperfusion with
RBC solution
n=7
Bulk RNAseq
n=7
Bulk RNAseq
n=7
Bulk RNAseq
n=7
Final cohort
n=5
Final cohort
n=6
Final cohort
n=6
