# Supplementary material for: A comparative analysis of acellular versus red cell-based subnormothermic machine perfusion in human kidney transplantation
Source: Am J Transplant. 2026 Nov;26(11):2511–23. doi: 10.1016/j.ajt.2026.04.014 (PMC13768858; doi:10.1016/j.ajt.2026.04.014)
Supplement: Supplementary file 2 [file mmc3.pdf]

A

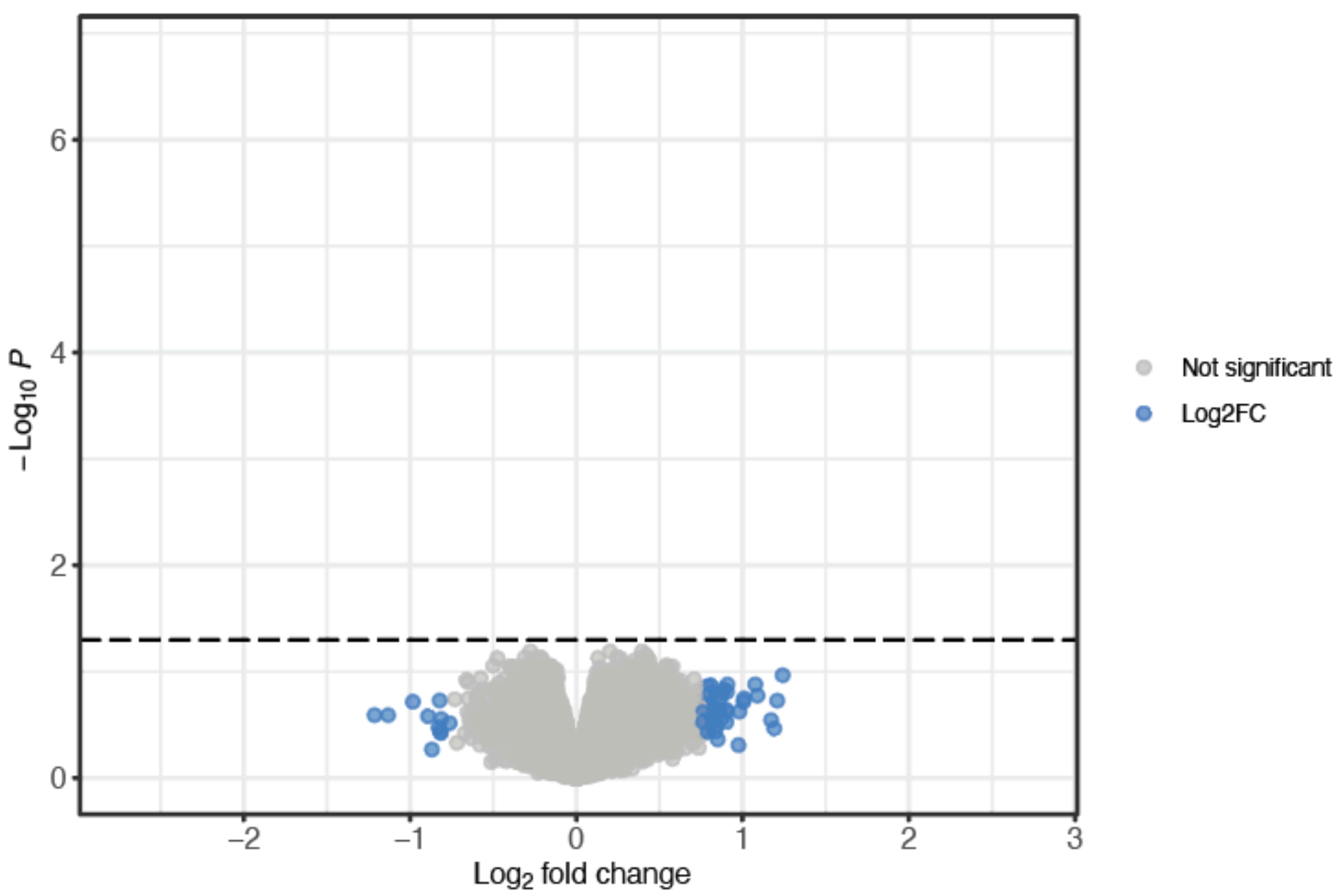

6
4
2
0
−Log10 P
−2
−1
0
1
2
3
Log2 fold change
Not significant
Log2FC
