# Supplementary material for: A comparative analysis of acellular versus red cell-based subnormothermic machine perfusion in human kidney transplantation
Source: Am J Transplant. 2026 Nov;26(11):2511–23. doi: 10.1016/j.ajt.2026.04.014 (PMC13768858; doi:10.1016/j.ajt.2026.04.014)
Supplement: Supplementary file 3 [file mmc4.pdf]

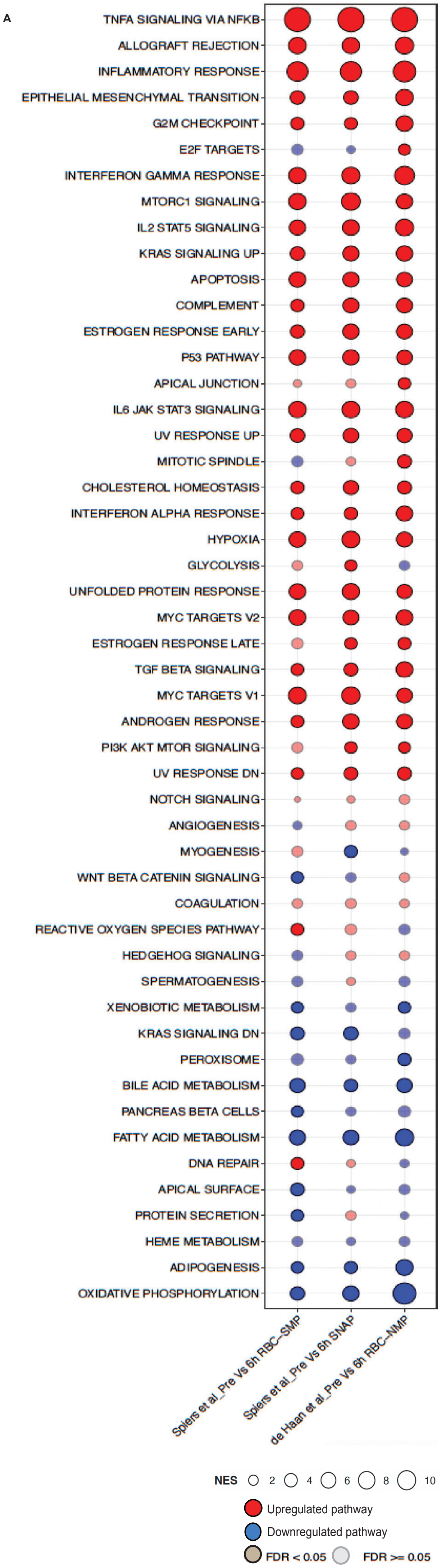

A
TNFA SIGNALING VIA NFKB
ALLOGRAFT REJECTION
INFLAMMATORY RESPONSE
EPITHELIAL MESENCHYMAL TRANSITION
G2M CHECKPOINT
E2F TARGETS
INTERFERON GAMMA RESPONSE
MTORC1 SIGNALING
IL2 STAT5 SIGNALING
KRAS SIGNALING UP
APOPTOSIS
COMPLEMENT
ESTROGEN RESPONSE EARLY
P53 PATHWAY
APICAL JUNCTION
IL6 JAK STAT3 SIGNALING
UV RESPONSE UP
MITOTIC SPINDLE
CHOLESTEROL HOMEOSTASIS
INTERFERON ALPHA RESPONSE
HYPOXIA
GLYCOLYSIS
UNFOLDED PROTEIN RESPONSE
MYC TARGETS V2
ESTROGEN RESPONSE LATE
TGF BETA SIGNALING
MYC TARGETS V1
ANDROGEN RESPONSE
PI3K AKT MTOR SIGNALING
UV RESPONSE DN
NOTCH SIGNALING
ANGIOGENESIS
MYOGENESIS
WNT BETA CATENIN SIGNALING
COAGULATION
REACTIVE OXYGEN SPECIES PATHWAY
HEDGEHOG SIGNALING
SPERMATOGENESIS
XENOBIOTIC METABOLISM
KRAS SIGNALING DN
PEROXISOME
BILE ACID METABOLISM
PANCREAS BETA CELLS
FATTY ACID METABOLISM
DNA REPAIR
APICAL SURFACE
PROTEIN SECRETION
HEME METABOLISM
ADIPOGENESIS
OXIDATIVE PHOSPHORYLATION
Spiers et al_Pre Vs 6h RBC-SMP
Spiers et al_Pre Vs 6h SNAP
de Haan et al_Pre Vs 6h RBC-NMP
NES 2 4 6 8 10
Upregulated pathway
Downregulated pathway
FDR < 0.05
FDR >= 0.05
