# Supplementary material for: A comparative analysis of acellular versus red cell-based subnormothermic machine perfusion in human kidney transplantation
Source: Am J Transplant. 2026 Nov;26(11):2511–23. doi: 10.1016/j.ajt.2026.04.014 (PMC13768858; doi:10.1016/j.ajt.2026.04.014)
Supplement: Supplementary file 4 [file mmc5.docx]

**Methods**

## **Sampling during machine perfusion**

Cortical wedge biopsies were taken at the end of cold storage, after 6 hours of subnormothermic perfusion and after four hours of red cell perfusate at 37^o^C. One half of each wedge was formalin-fixed (10% formaldehyde) and paraffin-embedded (FFPE) and one stored in RNA*later* for 24 hours prior to transfer to -80^o^C until analysed. Urine was sampled hourly and samples were centrifuged at 1600RPM for 10min at 4°C, the supernatant (bar 200uL above the buffy coat) was collected and snap frozen in liquid nitrogen then stored at -80°C until analysis.

### **Haematoxylin and eosin (H&E) staining and histological analysis**

Clinical grade H&E staining was completed by the Human Research Tissue Bank at Addenbrooke’s Hospital on 4μm FFPE sections. All H&E-stained sections were imaged using an Olympus IX81 inverted microscope with a 10x objective (Olympus Corporation, Tokyo, Japan). A minimum of five fields of view per section were imaged. A consultant renal transplant pathologist (AP) provided an injury report on representative sections from each timepoint of each experiment, blinded to perfusate allocation and perfusion timepoint.

### **Tissue RNA extraction**

For 7 kidney pairs, samples were thawed on ice, removed from storage solution and macerated on ice using a sterile scalpel. Macerated tissue was transferred to 700uL QIAzol Lysis Reagent (Qiagen, Hilden, Germany) and homogenised using a sterile pestle followed by 10x passages through a 20g sterile needle. 140uL of chloroform was added and sample shaken for 15 seconds by hand before being centrifuged at 12,000g for 15 minutes at 4°C. The clear upper supernatant (aqueous phase) containing RNA was taken forward and 1.5x the volume of 100% ethanol was added. This was then loaded onto Qiagen RNeasy Midi columns and total RNA extracted according to manufacturer’s instruction. RNA was eluted in 50uL of RNAse free water. DNA was removed on column using the Qiagen RNase-Free DNase set. Quality of RNA was assessed using nanodrop spectrophotometer and an RNA nano Bioanalyzer kit (Agilent Technologies, Palo Alto, CA, USA) using a Bioanalyzer 2100 (Agilent Technologies, Palo Alto, CA, USA). Samples with a RIN >6 were sent for sequencing.

### **Bulk RNA sequencing**

RNA was shipped on dry ice to Genewiz Azenta (Oxford, UK) for bulk RNA sequencing. Once received, RNA samples were quantified using Qubit 4.0 Fluorometer (Life Technologies, Carlsbad, CA, USA) and RNA integrity was checked with RNA Kit on Agilent 5300 Fragment Analyzer (Agilent Technologies, Palo Alto, CA, USA). rRNA depletion was performed using QIAGEN FastSelect rRNA HMR Kit (Qiagen, Hilden, Germany). RNA sequencing library preparation was performed with NEBNext Ultra II RNA Library Preparation Kit for Illumina following the manufacturer’s recommendations (NEB, Ipswich, MA, USA). Briefly, enriched RNAs were fragmented for 15 minutes at 94 °C. First strand and second strand cDNA were subsequently synthesized. cDNA fragments were end repaired, adenylated at 3’ends, and universal adapters were ligated to cDNA fragments, followed by index addition and library enrichment with limited cycle PCR. Sequencing libraries were validated using the Agilent Tapestation 4200 (Agilent Technologies, Palo Alto, CA, USA), and quantified using Qubit 2.0 Fluorometer (ThermoFisher Scientific, Waltham, MA, USA) as well as by quantitative PCR (KAPA Biosystems, Wilmington, MA, USA).

The sequencing libraries were multiplexed and loaded on the flowcell on the Illumina NovaSeq 6000 instrument according to manufacturer’s instructions. The samples were sequenced using a 2x150 Pair-End (PE) configuration v1.5. Image analysis and base calling were conducted by the NovaSeq Control Software v1.7 on the NovaSeq instrument. Raw sequence output (.bcl files) generated from Illumina NovaSeq was converted into fastq files and de-multiplexed using Illumina bcl2fastq program version 2.20. One mismatch was allowed for barcode matching.

#### **Transcriptomic analysis**

The raw mRNAseq samples were subjected to preprocessing comprising subsampling without replacement(22) to 75M reads to balance variable sequencing depth in samples. Trimming reads to 100 nucleotides (nts) reduced adapter contamination to within accepted levels (<5% across all samples). The proportion of retained biological signal, before and after each processing step, was assessed using fastQC(23), summarised using multiQC.(24) Poor quality samples, from a technical QC perspective, were designated if they contained a significantly higher overall GC%, or low numbers of unique reads indicating degradation, and subsequently excluded (n=5/42 samples, 11.9%). Mapping was performed using STAR (v2.7.4a)(25), with default parameters against vHg38 of the H sapiens genome. The quantification of genes, based on the H Sapiens gtf annotation was performed using featureCounts(26). Noise correction at expression-matrix level was performed using noisyR(27); the normalisation of gene expression was performed using quantile normalisation.(28) Differential expression analysis of paired samples was performed using DESeq2(29). A log_2_(FC) threshold cut off of 0.75 and adjusted p-value 0.05, using Benjamini-Hochberg multiple testing correction, were used to predict DE mRNA transcripts for subsequent analyses. For gene set enrichment analysis (GSEA), all differentially expressed genes were ranked by the inverse of the p value with the sign of the log fold-change, then ran against the Hallmark’s database within MSigDB(30), using the GSEA tool from the Broad Institute(31) with the pre-ranked option, and the following parameters: No_collapse, classic, default settings. Single sample GSEA (ssGSEA) was carried out using normalized counts from quantile normalization using the GenePattern platform(32).

**Transcriptomic Pathway Activity Scoring**

The gene sets used for scoring of glycolysis, fatty acid oxidation, gluconeogenesis, polyol metabolism, tricarboxylic acid cycle, glutamine synthesis, glutathione metabolism and osmotic stress, were obtained from Harmonizome(33).  For each sample the average expression of genes from the selected pathway gene set was calculated, and then subtracted with the average expression of another reference set of genes, in which the reference set was randomly sampled from all genes, and the subtracted value used as the “score”(34).

**De Haan *et al* publicly available transcriptomic data**

Raw data were downloaded from GEO GSE289688(11), annotated with relevant metadata provided by the authors, and re-normalised as outlined above.

**ELISA**

Assays were performed as directed by the manufacturer instructions. Urinary levels of kidney damage markers neutrophil gelatinase-associated lipocalin (NGAL) and liver fatty acid-binding protein (L-FABP) were measured using the Human Lipocalin-2/NGAL DuoSet ELISA kit (DY1757, R&D Systems) and the Human FABP1/L-FABP DuoSet ELISA kit (DY9465-05, R&D Systems). Urinary levels of proximal tubular endothelial cell damage marker insulin-like growth factor-binding protein 7 (IGFBP7) and distal tubular endothelial cell damage marker tissue inhibitor of metalloproteinases 2 (TIMP2) were measured using the Human IGFBP-rp1/IGFBP-7 DuoSet ELISA set (DY1334-05, R&D Systems) and Human TIMP-2 DuoSet ELISA (DY971, R&D Systems), respectively as per the manufacturers instructions. Absorbance was read at 450nm with 560nm correction on FLUOstar Omega Microplate Reader (BMG LABTECH GmbH) with FLUOstar OPTIMA software (BMG LABTECH GmbH).

## **Statistical analysis**

Continuous data were tested for normality with the Shapiro–Wilk test. Average values are presented as mean ± standard deviation (SD) for entries following a normal distribution and median with interquartile range (IQR) otherwise. Comparisons between three or more groups were performed using a mixed effects model or Kruskal–Wallis tests with Tukey’s or Dunn’s multiple comparisons corrections respectively. A two-tailed p-value of <0.05 is considered statistically significant. Analyses were conducted in GraphPad PRISM v10.1.1 (San Diego, CA, USA).
